# Sensitivity Deterioration of Free-Space Optical Coherent/Non-Coherent OOK Modulation Receiver by Ambient Light Noise

**DOI:** 10.3390/s23042140

**Published:** 2023-02-14

**Authors:** Weijie Ren, Jianfeng Sun, Haisheng Cong, Yuxin Jiang

**Affiliations:** 1 Key Laboratory of Space Laser Communication and Detection Technology, Shanghai Institute of Optics and Fine Mechanics, Chinese Academy of Sciences, Shanghai 201800, China; 2 Center of Materials Science and Optoelectronics Engineering, University of Chinese Academy of Sciences, Beijing 100049, China; 3 Laboratory of Space Laser Engineering, Shanghai Institute of Optics and Fine Mechanics, Chinese Academy of Sciences, Shanghai 201800, China

**Keywords:** OOK modulation, ambient light noise, coherent reception, non-coherent reception, noise power spectral density, sunlight coupling efficiency

## Abstract

In free-space optical (FSO) communication systems, on–off keying (OOK) is a widely used modulation format. Coherent and non-coherent OOK receivers with sensitivities of −54.60 dBm and −51.25 dBm, respectively, were built with a communication rate of 1 Gbit/s and a bit error rate of 10^−3^. In an FSO communication system, the parameters must be designed to ensure a sufficient link margin. In contrast to optical fiber systems, FSO systems have ambient light (AL) noise such as sunlight. The efficiency of sunlight coupling in the single-mode fiber (SMF) of the receivers was calculated in this study. For a signal light with AL, the change in the main components of noise and the sensitivity deterioration were theoretically analyzed and experimentally verified in conditions of coherent reception and non-coherent reception with a preamplifier. For coherent reception, the theoretical sensitivity deterioration results are consistent with the experimental results which indicate that coherent reception exhibits better anti-AL noise performance than non-coherent reception when the power spectral density of the AL is the same. Coherent and non-coherent receivers coupled with SMF can work in direct sunlight. When the receiver lens diameter is greater than 4.88 × 10^−4^ m, the anti-AL noise performance of the receiver can be improved by increasing the receiver lens diameter.

## 1. Introduction

Free-space optical (FSO) communication has unique advantages including a large bandwidth, an unlicensed spectrum, a high data rate, rapid deployment, and low power consumption [1], leading to extensive research and experiments. The European Space Agency (ESA) launched the Inter Satellite Link Experiment (SILEX) in 1990 [2]. A two-way communication laser link between the geostationary orbit satellite (ARTEMIS) and the low-orbit satellite (SPOT-4) was established in 2002; the communication format was on–off keying (OOK) [3]. In 2003, an optical payload (laser utilizing communication equipment (LUCE)) developed in Japan was installed in the ESA ground station; two-way communication experiments were conducted with the optical terminals on the ARTEMIS satellite; the uplink OOK rate was 49.37 Mbit/s [4]. In 2005, Japan’s OICETS satellite and ESA’s ARTEMIS satellite successfully conducted the first two-way optical communication between satellites in orbit [5]. In 2013, the lunar laser communication demonstration (LLCD) in the US achieved high-speed two-way laser communication between lunar orbiting satellites and the ground using the pulse position modulation (PPM) format [6,7,8]. In 2015, the Japan Aerospace Exploration Agency (JAXA) announced the next generation of Japan Data Relay Systems (JDRS), with optical communication using return to zero (RZ) differential phase shift keying (DPSK) and 50-Mbit/s OOK [9]. In 2016, the Shanghai Institute of Optics and Fine Mechanics conducted laser communication experiments using PPM for uplink communication [10].

In addition to large-scale experiments conducted by large institutions, researchers have conducted OOK experiments. In 1994, Thomas et al. theorized that an intensity-modulated coherent receiver was insensitive to laser line width [11]. In 2006, Becker et al. conducted a 10-Gbit/s OOK heterodyne communication experiment, achieving a sensitivity of −31 dBm [12]. In 2007, Wree et al. studied the noise performance of 10-Gbit/s OOK with heterodyne reception [13]. In 2015, Li et al. analyzed an OOK receiver with a Gaussian–Schell model laser [14]. In 2015, Li et al. analyzed the bit error rate (BER) of the OOK modulation scheme with non-coherent demodulation for space uplink optical communication systems [15]. In 2019, Parween et al. analyzed FSO communication using amplitude modulation (AM), OOK-RZ, and OOK-non return to zero (NRZ) [16]. In 2021, 10-Gbit/s OOK data were transmitted over 15 cm in free space to up to three receivers located in three different cards using a silicon integrated beam steering and broadcasting device [17]. The OOK system has also been validated using white or visible light [18,19].

The literature indicates wide use of OOK in FSO systems. It is important to study the factors that affect the sensitivity of OOK receivers. Researchers have conducted many simulations on the impact of atmospheric turbulence on OOK systems, providing some solutions [20,21,22,23,24]. In addition to atmospheric turbulence, ambient light (AL) is an important factor in OOK FSO systems. In 1996, Toyoshima et al. presented the results of an experiment to measure ambient radiation [25]. In 2014, Fu et al. summarized the characteristics of vacuum space environments [26]. In 2017, Lu et al. introduced a simulator that could simulate actual AL noise in a space environment [27]. In 2018, Farrell et al. discussed the expected AL sources and levels in an FSO system [28]. In 2021, Xu et al. proposed wavefront coherent compensation technology in direct sunlight in an FSO system [29]. In 2022, Giggenbach et al. summarized the basic relations of free-space avalanche photodiode (APD) receivers with an emphasis on optical low-earth orbit (LEO) downlinks and reported the dependence of the optimum multiplication factor on the AL [30].

In this AL research, theoretical derivation and experimental verification of the FSO receiver sensitivity deterioration caused by AL were not reported. Given the AL characteristics and the narrow optical bandwidth of the FSO receiver, it is considered that a broadband light source with a flat power spectral density (PSD) in the passband can be used as AL for theoretical analysis and experimental verification. AL noise can be expressed in the same form as amplified spontaneous emission (ASE) [31,32]. This study analyzes the reasons for the loss of signal-to-noise ratio (SNR) in coherent reception and non-coherent reception with a preamplifier when the OOK signal light contains AL and reports the single sideband PSD (SSPSD) of different noise. The SNR loss in dB represents the sensitivity deterioration. With coherent reception, and with an increase in the PSD of the AL noise, the main component of the noise changes from shot noise to AL–local oscillator (LO) beat noise. The theoretical calculation results are consistent with the experimental results. The sensitivity deterioration of the coherent and non-coherent receivers was measured experimentally in AL with different PSDs; curves in two conditions of sensitivity deterioration vs. AL PSD were obtained. It was demonstrated that a coherent OOK receiving system has better anti-AL performance than a non-coherent OOK receiving system. AL intensity in the experiment was evaluated in terms of W/Hz. The design flow of the FSO system and calculation of the link margin were presented; the direct sunlight coupling efficiency was calculated, and the sunlight power coupled to the receiver single-mode fiber (SMF) for the specified optical bandwidth was analyzed. With the sun shining directly on the OOK receiver, the receiver optical bandwidth was 2 nm; the receiver lens diameter was 20 cm; the signal light had the optimum coupling efficiency, and the optical power coupled to the SMF was 16.96 nW. With this optical power, the PSD of sunlight can be calculated as $6.84\times 10^{-20}\;W/\mathrm{Hz}$. According to the curve of sensitivity deterioration vs. PSD of AL noise measured in the experiment, the sensitivity deterioration by direct sunlight in coherent reception and non-coherent reception was 1.15 dB and 2.60 dB, respectively. This indicates that the FSO OOK system can operate in direct sunlight and sunlight power entering the SMF does not increase with an increase in the receiver lens diameter within a certain range. Thus, AL noise can be controlled by increasing the receiver lens diameter, which is significant in FSO system research.

The remainder of this study is organized as follows. In Section 2, the principle of OOK coherent/non-coherent reception is introduced, and the corresponding high-sensitivity OOK receiver is built to prepare for the study of sensitivity deterioration caused by the AL. Section 3 introduces the link margin of FSO system and the coupling efficiency of signal light and sunlight to the receiver SMF. Section 4 theoretically analyzes the influence of AL on OOK coherent/non-coherent reception. Section 5 introduces the experimental research on the influence of AL on coherent/non-coherent reception of OOK signal light. Section 6 presents the summary and conclusions.

## 2. Principle and Device of Coherent/Non-Coherent OOK Reception without AL

**Figure 1:**
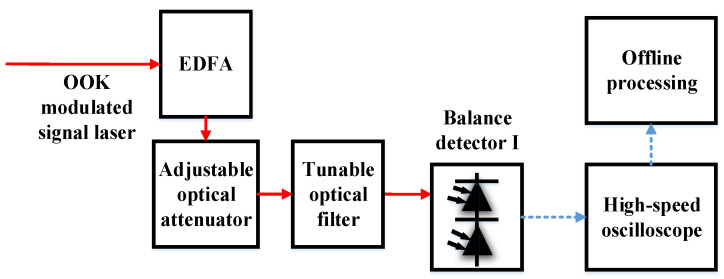
Experimental structure of non-coherent OOK receiver.

In order to study the sensitivity deterioration caused by AL noise, high-sensitivity coherent and non-coherent OOK receiving devices were designed and completed. The information demodulation of the receiver in the FSO OOK system is primarily based on direct detection (DD). APDs are often used in DD receivers for optical-to-electrical conversion [30,33]. However, the sensitivity of a DD system using an APD is inferior to that of a positive intrinsic negative photodiode (PIN) and a preamplifier. Thus, the latter was selected as the non-coherent reception mode in this study. The preamplifier was an erbium-doped fiber amplifier (EDFA) as the central wavelength of the signal light was 1555.74 nm. The EDFA had a gain of 30 dB and a noise figure of 5.25 dB. A narrowband optical filter is an important device for ensuring high sensitivity in a DD system using preamplifiers. In DD reception experiments, we used a tunable filter and set the full width at half maximum (FWHM) to 0.8 nm, which means the FWHM is from 1555.34 nm to 1556.14 nm. The reason for choosing 0.8 nm is that it is one of the standard wavelengths of dense wavelength division multiplexing (DWDM) and can ensure that the signal light does not appear outside the passband of the optical filter through the influence of Doppler frequency shift. The tunable optical filter in this experiment was the BVF-200 from Alnair Labs. A structural diagram of the non-coherent receiver is shown in Figure 1.

**Figure 2:**
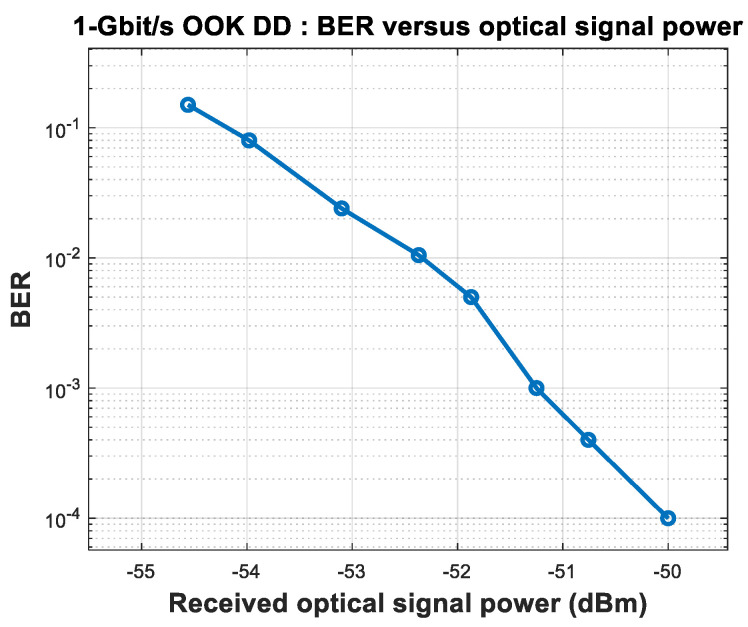
Curve between BER and received signal power of OOK DD with a communication rate of 1 Gbit/s.

In the experiment, the signal light entered the light input of the balance detector (BD), which is equivalent to a PIN, after the tunable optical filter. In this experiment, the communication rate was 1 Gbit/s; we obtained the curve between the BER and optical signal power when receiving non-coherently, as shown in Figure 2. When the BER is 10^−3^, the optical signal power was −51.25 dBm. The sampling rate of the high-speed oscilloscope was 5 GSa/s, and the channel analog bandwidth was set to 1 GHz. The offline processing (Figure 1) included low-pass filtering, 10× resampling, time synchronization, and threshold decisions.

**Figure 3:**
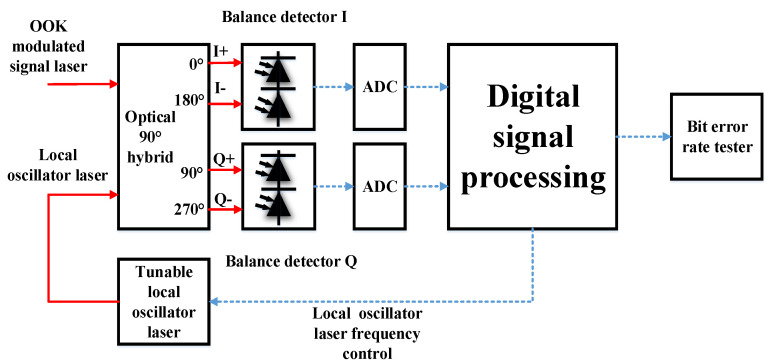
Experimental structure of coherent OOK receiver.

The experimental results for non-coherent reception without AL are shown in Figure 2. The experimental structure for coherent reception of OOK signal light is shown in Figure 3.

For the coherent reception of OOK, the best LO power was determined to be 12.8 mW by calculating the SNR of the beat frequency signals using the cross-correlation method. The OOK format signal light and the LO light enter the optical 90° hybrid, and the hybrid outputs four channels of light. Two lights from the in-phase (I) branch enter one BD and two lights from the quadrature (Q) branch enter the other BD. The BD was the same as the BD used for non-coherent reception. The voltage signals generated by the four output lights are presented as [34,35,36]

(1) $$U_{0}\left(t\right)=Rr\left[\frac{1}{2}{\left(s(t)\right)}^{2}\left(1-k_{S}\right)P_{S}+\frac{1}{2}\left(1-k_{LO}\right)P_{LO}+\sqrt{1-k_{S}}\sqrt{1-k_{LO}}\sqrt{P_{LO}P_{S}}s\left(t\right)\cos\left(\omega_{S}t-\omega_{LO}t+\varphi_{S}\left(t\right)-\varphi_{LO}\left(t\right)\right)\right]+n_{0}\left(t\right),$$

(2) $$U_{90}\left(t\right)=Rr\left[\frac{1}{2}{\left(s(t)\right)}^{2}k_{S}P_{S}+\frac{1}{2}k_{LO}P_{LO}+\sqrt{k_{S}}\sqrt{k_{LO}}\sqrt{P_{LO}P_{S}}s\left(t\right)\sin\left(\omega_{S}t-\omega_{LO}t+\varphi_{S}\left(t\right)-\varphi_{LO}\left(t\right)\right)\right]+n_{90}\left(t\right),$$

(3) $$U_{180}\left(t\right)=Rr\left[\frac{1}{2}{\left(s(t)\right)}^{2}\left(1-k_{S}\right)P_{S}+\frac{1}{2}\left(1-k_{LO}\right)P_{LO}-\sqrt{1-k_{S}}\sqrt{1-k_{LO}}\sqrt{P_{LO}P_{S}}s\left(t\right)\cos\left(\omega_{S}t-\omega_{LO}t+\varphi_{S}\left(t\right)-\varphi_{LO}\left(t\right)\right)\right]+n_{180}\left(t\right),$$

(4) $$U_{270}\left(t\right)=Rr\left[\frac{1}{2}{\left(s(t)\right)}^{2}k_{S}P_{S}+\frac{1}{2}k_{LO}P_{LO}-\sqrt{k_{S}}\sqrt{k_{LO}}\sqrt{P_{LO}P_{S}}s\left(t\right)\sin\left(\omega_{S}t-\omega_{LO}t+\varphi_{S}\left(t\right)-\varphi_{LO}\left(t\right)\right)\right]+n_{270}\left(t\right),$$

where R is the detector responsivity; r is the detector transimpedance; s(t) is the modulation term of the communication signal; s(t)=0 or 1; $k_{S}$ and $k_{LO}$ are the signal light splitting ratio and the LO light splitting ratio of the optical 90° hybrid, respectively, both of which are 0.5 in this study; $P_{S}$ and $P_{LO}$ are the signal optical power and LO optical power, respectively; $\omega_{S}$ and $\omega_{LO}$ are the signal optical angular frequency and LO optical angular frequency, respectively; $\varphi_{S}\left(t\right)$ and $\varphi_{LO}\left(t\right)$ are the random phase of the signal optical carrier and the random phase of the LO optical carrier, respectively; $n_{0}\left(t\right)$, $n_{180}\left(t\right)$, $n_{90}\left(t\right)$, and $n_{270}\left(t\right)$ are additive noise of the four voltage signals, respectively. With no preamplifier and LO power of 12.8 mW, the four additive noise signals are mainly LO shot noise.

After the two BDs are completely subtracted to eliminate the communication square term and direct current (DC) term, I and Q voltage signals are obtained as follows with $k_{S}=k_{LO}=0.5$ [37]

(5) $$U_{I}\left(t\right)=U_{0}\left(t\right)-U_{180}\left(t\right)=As\left(t\right)\cos\left(\Delta \varphi \left(t\right)\right)+n_{I}\left(t\right),$$

(6) $$U_{Q}\left(t\right)=U_{90}\left(t\right)-U_{270}\left(t\right)=As\left(t\right)\sin\left(\Delta \varphi \left(t\right)\right)+n_{Q}\left(t\right),$$

where $A=Rr\sqrt{P_{S}P_{LO}}$; $\Delta \varphi \left(t\right)=\omega_{S}-\omega_{LO}t+\varphi_{s}\left(t\right)-\varphi_{LO}\left(t\right)$; $n_{I}\left(t\right)=n_{0}\left(t\right)-n_{180}\left(t\right)$ and $n_{Q}\left(t\right)=n_{90}\left(t\right)-n_{270}\left(t\right)$; $n_{I}\left(t\right)$ and $n_{Q}\left(t\right)$ are I and Q noise, respectively.

After synchronous sampling, the I and Q signals were complexly transformed, expressed as

(7) $$U_{COOOK}\left[k\right]=U_{I}\left[k\right]+j\times U_{Q}\left[k\right]=As\left[k\right]\exp\left(j\times \Delta \varphi \left[k\right]\right)+n_{I}\left[k\right]+j\times n_{Q}\left[k\right],$$

where k is the index of the sampled signal and $j=\sqrt{-1}$. The modulus of the sampled complex signal is expressed as

(8) $$U_{ABSOOK}\left[k\right]={\left(real\left(U_{COOOK}\left[k\right]\right)\right)}^{2}+{\left(imag\left(U_{COOOK}\left[k\right]\right)\right)}^{2}\approx {\left(As\left[k\right]\right)}^{2},$$

where real(·) represents the real part of the complex signal; and imag(·) represents the imaginary part of the complex signal. Equation (9) can be obtained by taking the square root of the signal.

(9) $$U_{EXTOOK}\left[k\right]=\sqrt{U_{ABSOOK}\left[k\right]}=As\left[k\right],$$

**Figure 4:**
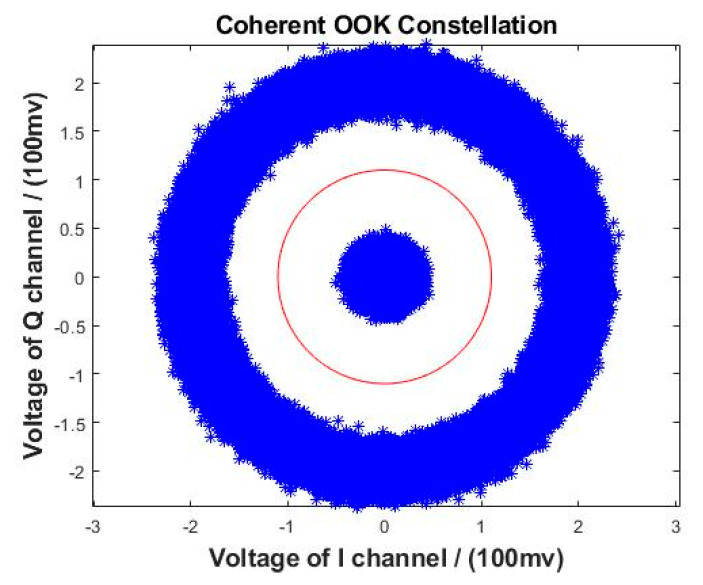
Constellation of OOK for coherent reception (simulated SNR: 20 dB).

As s[k]=0 or 1, the coherent demodulation of the OOK signal can be determined only by selecting an appropriate threshold. The communication rate was also 1 Gbit/s in the coherent OOK reception experiment to ensure consistency with non-coherent reception. The two analog-to-digital converters (ADC) in Figure 3 were replaced by a high-speed oscilloscope (Figure 1); the oscilloscope settings were consistent with those during non-coherent reception. The two oscilloscope channels synchronously sampled the output signals of the two BDs. Before receiving the signal, the LO frequency was adjusted such that the frequency difference between the LO light and the signal light was far less than the communication rate; thus, intradyne detection was implemented. One advantage of this method is that it reduces the electrical bandwidth requirement of the detector. To reduce the electrical bandwidth, Wu et al. presented a spectrally sliced heterodyne coherent receiver with halved electrical bandwidth [38]. The constellation of coherent reception of the OOK signal is shown in Figure 4.

**Figure 5:**
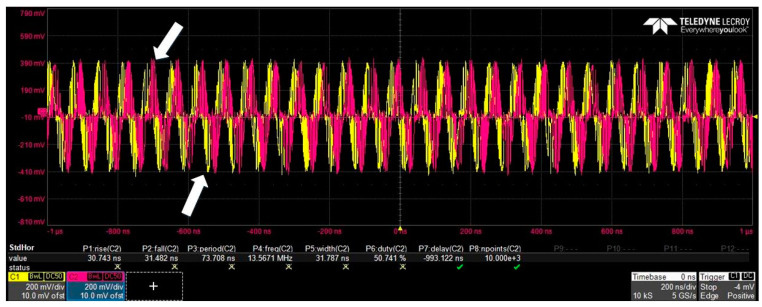
I and Q signals of coherent OOK (down arrow indicates to I-channel signal, up arrow indicates Q-channel signal).

The BDs used in the experiment were PDB780CAC (THORLABS); the typical common mode rejection ratio (CMRR) of the BDs was greater than 25 dB, meeting the experimental requirements. The optical 90° hybrid was COH24 (Kylia). The oscilloscope was a Lab Master MCM-Zi-A (TELEDYNE LECORY). The I and Q signals of the coherent OOK before algorithm recovery are shown in Figure 5.

The key experimental parameters in this paper are presented in Table 1.

OOK coherent demodulation is asynchronous reception; the relationship between the BER and the optical signal power under the shot noise limit is expressed as [39]

(10) $$BER_{COOOK}=0.5\times \exp\left(-\frac{\eta P_{S}}{2hvR_{b}}\right),$$

where η is the quantum efficiency, which equals 1 in the ideal state; *hv* is the photon energy; $P_{S}$ is the signal light receiving power; and $R_{b}$ is the communication rate here. Carrier recovery is required for the coherent reception of phase-shift keying (PSK) signals [40]. Carrier recovery is not required for coherent reception of OOK signals, an advantage of OOK coherent reception.

**Figure 6:**
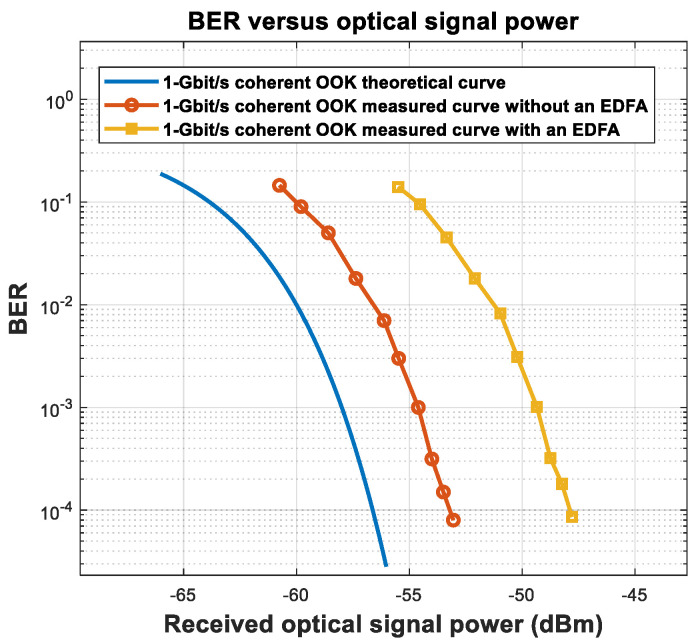
Measured and theoretical curves of coherent OOK BER and received signal power with a communication rate of 1 Gbit/s.

By continuously reducing the optical signal power, the relationship between the BER and the optical signal power for OOK coherent reception is obtained, as shown in Figure 6.

When the OOK signal is coherently received and the BER is 10^−3^, the optical signal power is −54.60 dBm, with a loss of 3.3 dB compared to the shot noise limit. The main reason for the loss is the responsivity of the detector (0.85 A/W), which causes a loss of 1.67 dB compared with a responsivity of 1.25 A/W with ideal quantum efficiency. There is a connecting flange for the output of the hybrid to enter the detector, which causes a loss of 0.3 dB. The remaining 1.33 dB may be caused by the transmissivity of the detector window, imperfect heterodyne efficiency, or ADC quantization loss. When the BER is 10^−3^, the loss can be reduced through forward error correction (FEC) [41].

**Figure 7:**
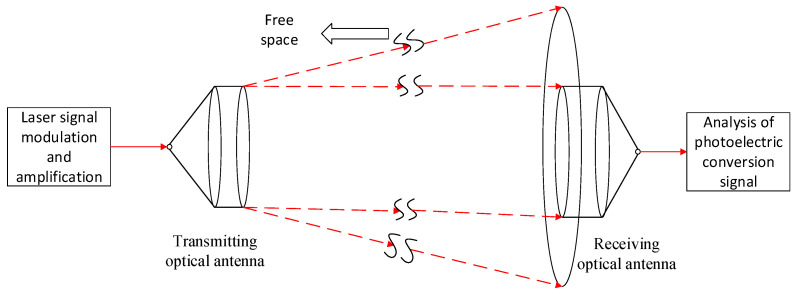
Simple block diagram of FSO communication.

For OOK coherent demodulation, an EDFA can be used to pre-amplify the signal light, with the advantage of reducing the LO power requirement. However, after the EDFA is added, the main noise component changes from LO shot noise to ASE–LO beat noise. Experimental measurement shows that the optical signal power is −49.3 dBm when the BER is 10^−3^ when using an EDFA, decreasing the sensitivity by 5.3 dB compared with reception without an EDFA, as shown in Figure 7. To ensure high sensitivity, we choose not to add an EDFA for coherent reception of the OOK signal. So far, the experimental device for verifying the influence of AL noise has been completed.

## 3. Link Margin of FSO System and SMF Coupling Efficiency of Signal Light and Sunlight

For better integration in practice, an actual FSO communication system was considered; the link margin was calculated to determine whether the receiver sensitivity met the requirements. In this study, a satellite–ground laser communication system was considered. The coupling efficiency of the signal light and AL to the SMF is important. In this study, sunlight was considered as major AL; the analysis is presented as follows.

A simple block diagram of FSO communication is shown in Figure 7. The differences between an FSO system and an optical fiber system are mainly as follows. An FSO system includes received optical power fluctuation, Doppler frequency shift, tracking error, platform jitter, and coupling of space light to receiver SMF. The main difference considered in this paper is the coupling of space light to a receiver SMF.

In this design, the laser wavelength λ was 1555.74 nm; the effective transmitting aperture $D_{T}$ was 20 cm; the corresponding beam divergence angle was $\theta_{T}=1.22\lambda /D_{T}=9.49\;\left(\mu \mathrm{rad}\right)$; and the transmitting antenna gain was $G_{T}=10\log10\left(16/{\left(\theta_{T}\right)}^{2}\right)=112.49\;\left(\mathrm{dB}\right)$. For the receiving antenna gain, in satellite–ground laser communication, the communication distance is long and the spot size reaching the receiving terminal is much larger than the receiving antenna aperture; thus, it can be considered that the beam reaching the receiving antenna is a plane wave.

When the communication distance z was 40,000 km; the free space loss was $\mathrm{FSL}=10\times \log10\left({\left(\lambda /4\pi z\right)}^{2}\right)=-290.19\;\left(\mathrm{dB}\right)$. When the effective aperture of the receiver $D_{R}$ was 20 cm, the receiving antenna gain was $G_{R}=10\times \log10\left({\left(\pi D_{R}/\lambda \right)}^{2}\right)=112.12\;\left(\mathrm{dB}\right)$.

Assuming that the transmission efficiency loss was 1.5 dB and the reception efficiency loss was 3 dB, the total link loss was 290.19+1.5+3−112.49−112.12=70.08 (dB), regardless of the atmosphere, alignment, and coupling losses. When the transmission power was 33 dBm, the residual power was −37.08 dBm. When the BER was 10^−3^, the link margins for coherent reception and non-coherent reception were 17.52 dB and 14.17 dB, respectively.

In this study, we considered the coupling of the signal light and AL to the receiver SMF. In an FSO system, when a signal light enters the receiver, it can be regarded as a plane wave. When there is no alignment error, the coupling efficiency of the signal light to SMF can be expressed as [42]

(11) $$\eta =2{\left[\frac{\exp\left(-\beta^{2}\alpha^{2}\right)-\exp\left(-\beta^{2}\right)}{\beta \sqrt{1-\alpha^{2}}}\right]}^{2},$$

where β is the coupling parameter, $\beta =\pi D_{R}\omega_{0}/2\lambda f$; $D_{R}$ is the receiver lens diameter or the effective aperture of the receiver; $\omega_{0}$ is the mode field radius of the SMF, $\omega_{0}=5\;\mu m$ in this study; f is the focal length of the receiving lens; α is the linear central obstruction, α=0 in this study. When β=1.12 was used, the optimum coupling efficiency of the 1555.74 nm signal light was 0.81 [43]. To achieve the optimum coupling efficiency, the focal length of the receiver lens should be 0.90 m when the receiver lens diameter is 20 cm. In other words, each receiver lens diameter can uniquely determine a lens focal length so that 1550.74 nm signal light has the optimum coupling efficiency to an SMF. When light from different directions shines on the lens, the coupling efficiency is expressed as [44]

(12) $$\eta \left(\theta_{e}\right)=\frac{{\left|\int_{0}^{1}\sqrt{8\pi }\beta \exp\left(-\beta^{2}\rho^{2}\right)J_{0}\left(2\beta \frac{f}{\omega_{0}}\theta_{e}\rho \right)\rho d\rho \right|}^{2}}{\pi },$$

where $\theta_{e}$ is the angle between the incident direction of light and the optical axis of the lens or the direction angle; ρ is the normalized radial position; and $J_{0}\left(\cdot \right)$ represents the zero-order Bessel function. In this study, light from different directions can be regarded as plane waves.

By adding coupling loss and atmospheric transmission loss, the final link margin of an FSO receiving system can be calculated.

The signal light can be considered as a plane wave, the sunlight cannot be considered as a plane wave. The average distance between the sun and the earth is $D_{S-E}=1.496\times 10^{11}\;\left(m\right)$; the sun diameter is $R_{S}=1.39\times 10^{9}\;\left(m\right)$ and the angle of the sun to the FSO receiver is approximately ±4.65 mrad, which is greater than the receiving field angle of the FSO receiver system. Thus, the sun was regarded as a surface light source. The solid angle of the sun disk is $\Omega_{S}=\pi R_{S}^{2}/D_{S-E}^{2}\;\left(\mathrm{sr}\right)$. According to the Stefan–Boltzmann law [45], the radiation force of the sun is $E_{S}=\sigma T_{S}^{4}\;\left({W/m}^{2}\right)$, where σ is the Boltzmann constant and $T_{S}$ is the effective temperature of the solar surface, in Kelvin. The solar radiation intensity in the unit solid angle on the surface of the vertical solar ray at the upper boundary of the atmosphere of the earth is $I_{S}=E_{S}/\pi \;\left(W/\left(m^{2}\times \mathrm{sr}\right)\right)$. The solar constant refers to the solar radiation energy received by the upper boundary surface of the atmosphere perpendicular to the solar rays at the average distance between the sun and the earth in unit time and unit area. Thus, the theoretical calculation of the solar constant is written as

(13) $$I_{SC}=I_{S}\Omega_{S}=\frac{\sigma T_{S}^{4}}{\pi }\frac{\pi R_{S}^{2}}{D_{S-E}^{2}},$$

When $T_{S}=5766\;K$ and the solar constant is $1353{\;W/m}^{2}$.

**Figure 8:**
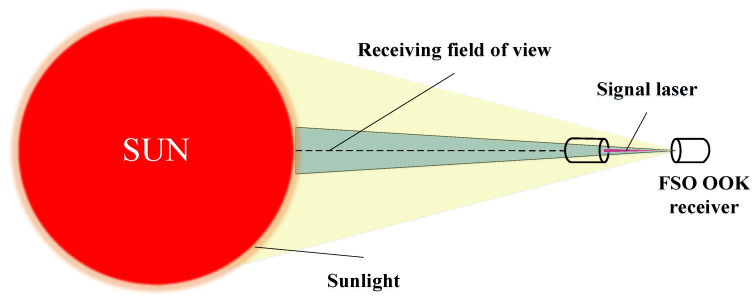
Diagram of direct sunlight on FSO receiver.

The solar constant was approximately $1367{\;W/m}^{2}$ and the energy in the full bandwidth spectrum, measured by an artificial satellite. In this study, the measured value of the solar constant was used for calculation. The laser wavelength used was 1555.74 nm, and the optical bandwidths of the coherent and non-coherent receiving systems were 2 nm and 0.8 nm, respectively. The solar irradiance at this wavelength was $267\;W/\left(m^{2}\times \mu m\right)$. A diagram of direct sunlight on the FSO receiver is shown in Figure 8.

The total power of sunlight on the receiver lens is expressed as

(14) $$P_{sunlens}=E_{sun}\times \pi {\left(D_{r}\right)}^{2}/4\times B_{o},$$

where $E_{sun}$ is the irradiance of sunlight at the wavelength of the signal light and $B_{o}$ is the optical bandwidth of the receiver. To ensure that the signal light has the optimum coupling efficiency, β=1.12 should be satisfied; each receiver lens diameter corresponds to an optimal lens focal length.

In this paper, it is considered that the sun directly irradiates the receiver lens. When the direction angle of incident light multiplied by the lens focal length is greater than twice the mode field radius of the SMF, the focused Airy spot will not appear in the mode field range of the SMF optical field; that is, the coupling efficiency is zero. The sun is a surface light source, which can be considered as a disk when it shines directly. Because the distance between the sun and the receiver is far enough, the light from each point of the solar disk can be regarded as a plane wave when it reaches the receiver. Each point of the solar disk has a coupling efficiency $\eta \left(\theta_{e},\phi \right)$, where $\theta_{e}$ is the angle between the line connecting the point and the receiver and the line connecting the solar disk center and the receiver, the direction angle. The range of $\theta_{e}$ is ±4.65 mrad. With the center of the solar disk as the origin, the line connecting the center of the disk and the rightmost point of the solar disk from the perspective of the receiver is the x-axis to establish a rectangular coordinate system; ϕ is the angle between the line connecting the point and the center of the disk and the positive direction of the x-axis. The range of ϕ is [0 rad,2π rad]. When $\theta_{e}$ is fixed and ϕ changes from 0 rad to 2π rad, the coupling efficiency of these points on the solar disk are the same. That is, in the solar disk, the points on the circle with the center of the disk as the center have the same coupling efficiency. In this study, we call the coupling efficiency on different circles as the integral coupling efficiency, which is only related to the direction angle $\theta_{e}$. Thus, AL generated by the sun with the same direction angle has the same coupling efficiency to the receiver SMF.

**Figure 9:**
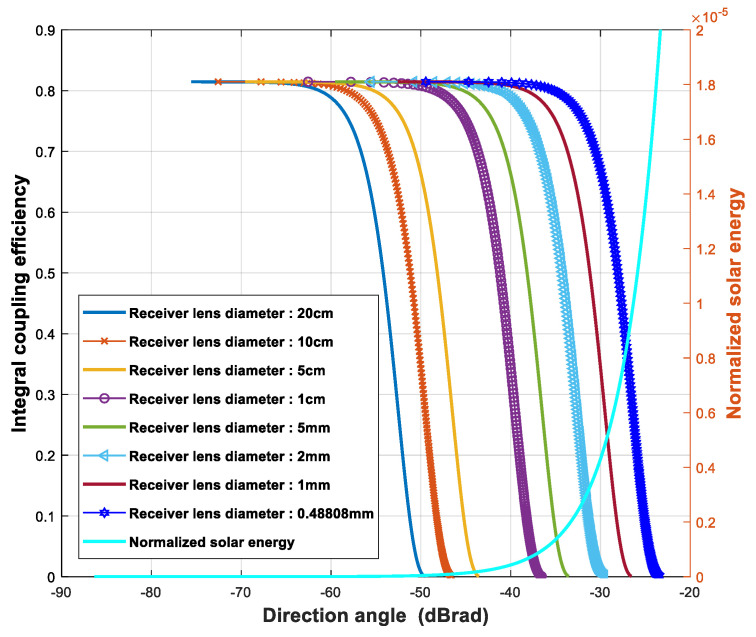
Curves of integral coupling efficiency vs. AL direction angle for different receiver lens diameters and normalized sunlight energy on receiver lens vs. AL direction angle.

In order to ensure the optimum coupling efficiency of signal light, β=1.12 should be met. When the receiver lens diameters were 20 cm, 10 cm, 5 cm, 1 cm, 5 mm, 2 mm, 1 mm, and 0.488 mm, respectively, the lens focal lengths corresponding to these receiver lens diameters were 0.90 m, 0.81 m, 0.68 m, 0.45 m, 0.23 m, 0.18 m, 0.14 m, and 0.09 m, respectively. According to Equation (12), the relationship curve between the integral coupling efficiency and the direction angle can be plotted for different receiver lens diameters as shown in Figure 9. The y-axis of these curves is the left y-axis in Figure 9. When the receiver lens diameter is 0.488 mm and the integral coupling efficiency becomes zero for the first time, the corresponding direction angle is exactly equal to 4.65 mrad, which is half of the angle of the sun to the FSO receiver. The shape of the solar disk is completely symmetrical; thus, we can only consider the positive direction angle. In other words, if the receiver lens diameter is less than or equal to 0.488 mm, the integral coupling efficiency of sunlight in the full-range direction angle is greater than zero, which means that 0.488 mm is a critical value.

The relationship curve between the normalized solar energy and the direction angle is also shown in Figure 9. The y-axis of this curve is the right y-axis in Figure 9. In order to facilitate the drawing, the horizontal axis was expressed by logarithmic calculation. The logarithm value corresponding to 4.65 mrad was −23.33 dBrad. When the relationship curve between the normalized energy and the sunlight direction angle was simulated on the right side of Figure 9, the simulation interval of the sunlight direction angle was 4.65 × 10^−8^ rad. The sum of the values corresponding to all points of different direction angles in the right curve was 1, that is, the sum of the sunlight energy in all direction angles.

When the receiver lens diameter is determined, there is a unique receiver lens focal length that can meet β=1.12. Thus, when the receiver lens diameter is given, the change in the integral coupling efficiency with the direction angle can be expressed as $\eta_{diameter}\left(\theta_{e}\right)$, which is consistent with the curves on the left of Figure 9. First, calculate the volume of the rotating body obtained by rotating $\eta_{diameter}\left(\theta_{e}\right)$ around the left y-axis in Figure 9. Here, we only take the positive part of $\theta_{e}$, thus, the range of $\theta_{e}$ is 0 to 4.65 mrad. The area corresponding to the full range of solar direction angle is $\pi {\left(4.65\times 10^{-3}\right)}^{2}$. The average coupling efficiency of sunlight is obtained by dividing the volume of the rotating body by $\pi {\left(4.65\times 10^{-3}\right)}^{2}$. Thus, the average coupling efficiency of sunlight to the receiver SMF with a certain receiver lens diameter can be expressed as

(15) $$\eta_{average(diameter)}=\frac{2\pi \int_{0}^{4.65\times 10^{-3}}\theta_{e}\times \eta_{diameter}\left(\theta_{e}\right)d\theta_{e}}{\pi {\left(4.65\times 10^{-3}\right)}^{2}},$$

**Figure 10:**
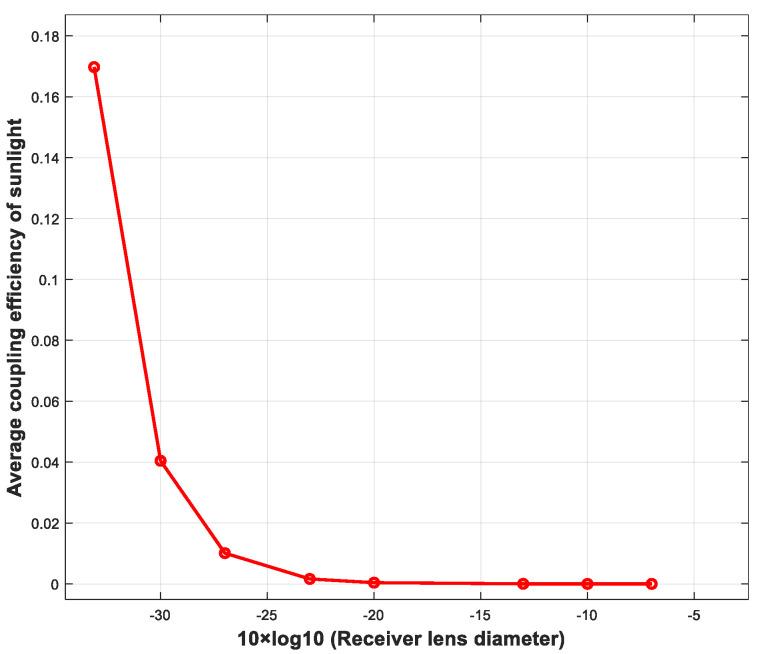
Curve of average coupling efficiency of sunlight vs. receiver lens diameter (receiver lens diameter with unit of meter).

In this study, the numerical integration method was used to calculate the average sunlight coupling efficiency of the SMF for different receiver lens diameters with Equation (15), as shown in Figure 10. When the receiver lens diameter is 20 cm, the average sunlight coupling efficiency of the SMF is 1.01 × 10^−6^.

The sunlight power coupled into the SMF with a certain receiver lens diameter is calculated using Equation (16).

(16) $$P_{sunSMF\left(diameter\right)}=P_{sunlens}\times \eta_{averge\left(diameter\right)},$$

According to the calculation in Equation (16), when the receiver lens diameters were the corresponding values in Figure 9 and the receiver optical bandwidth was 2 nm, the direct sunlight power coupled into the receiver SMF was 16.96 nW; the PSD of the sunlight coupled into the receiver SMF was $6.84\times 10^{-20}\;W/\mathrm{Hz}$ with different receiver lens diameters, as long as the receiver lens diameter was greater than 0.488 mm. In this condition, as the receiver lens diameter increases, the receiver gain increases, which is equivalent to an increase in the signal light power while the sunlight power remains unchanged. Thus, the sensitivity deterioration by sunlight noise can be suppressed by increasing the receiver lens diameter.

Although the coupling efficiency is also a function of the wavelength, the bandwidth of sunlight becomes very narrow after passing through the optical filter. The coupling efficiency was considered to be the same in this narrow wavelength range.

## 4. Theoretical Analysis of the Influence of AL on Coherent/Non-Coherent OOK Reception

When the influence of AL on the OOK coherent receiver was considered, the total electric field of the OOK signal light and AL entering the coherent receiving system is expressed as

(17) $$E\left(t\right)=s\left(t\right)\sqrt{2P_{S}}\cos\left(\omega_{S}t+\varphi_{S}\left(t\right)\right)+\sum_{i=-\left(B_{o}/2\delta v\right)}^{\left(B_{o}/2\delta v\right)}\sqrt{2S_{aln}\delta v}\cos\left[\left(\omega_{S}+2\pi i\delta v\right)t+\varphi_{i}\right],$$

where $S_{aln}$(W/Hz) is the PSD of the AL noise, which is a function of frequency and is considered to be the same in the optical bandwidth; $B_{o}$ (Hz) is the FWHM of the optical filter, the FWHM of the optical filter is the same as the optical bandwidth in this study; δv(Hz) is the subdivided frequency interval; i represents the serial number of each frequency interval; $\varphi_{i}$ represents the random phase at each frequency. The first term in Equation (17) is the signal light and the second term is the AL. In this study, it is considered that the PSD of the AL is flat within the optical bandwidth of the system.

The LO light can be expressed as

(18) $$E_{LO}\left(t\right)=\sqrt{2P_{LO}}\cos\left(\omega_{LO}t+\varphi_{LO}\left(t\right)\right),$$

At this time, the four light outputs of the optical 90° hybrid enter the balance detectors; all photogenerated currents can be expressed as follows after removing the DC term and sum frequency term. Compared with Equations (1) to (4), the change in the following expression is only because the AL is added to the signal light.

(19) $$i_{0}\left(t\right)=s^{2}\left(t\right)R\frac{P_{S}}{4}+i_{aln-aln}\left(t\right)+s\left(t\right)R\frac{\sqrt{P_{S}P_{LO}}}{2}\cos\left(\Delta \omega t+\Delta \varphi \right)+i_{S-aln}\left(t\right)+i_{Ialn-LO}\left(t\right)+n_{0c}\left(t\right),$$

(20) $$i_{90}\left(t\right)=s^{2}\left(t\right)R\frac{P_{S}}{4}+i_{aln-aln}\left(t\right)+s\left(t\right)R\frac{\sqrt{P_{S}P_{LO}}}{2}\sin\left(\Delta \omega t+\Delta \varphi \right)+i_{S-aln}\left(t\right)+i_{Qaln-LO}\left(t\right)+n_{90c}\left(t\right),$$

(21) $$i_{180}\left(t\right)=s^{2}\left(t\right)R\frac{P_{S}}{4}+i_{aln-aln}\left(t\right)-s\left(t\right)R\frac{\sqrt{P_{S}P_{LO}}}{2}\cos\left(\Delta \omega t+\Delta \varphi \right)+i_{S-aln}\left(t\right)-i_{Ialn-LO}\left(t\right)+n_{180c}\left(t\right),$$

(22) $$i_{270}\left(t\right)=s^{2}\left(t\right)R\frac{P_{S}}{4}+i_{aln-aln}\left(t\right)-s\left(t\right)R\frac{\sqrt{P_{S}P_{LO}}}{2}\sin\left(\Delta \omega t+\Delta \varphi \right)+i_{S-aln}\left(t\right)-i_{Qaln-LO}\left(t\right)+n_{270c}\left(t\right),$$

The specific expressions in Equations (19)–(22) are expressed as

(23) $$i_{aln-aln}\left(t\right)=R\frac{S_{aln}\delta v}{4}\sum_{i=-M}^{M}\sum_{j=-M}^{M}\cos\left(2\pi \left(i-j\right)\delta vt+\varphi_{i}-\varphi_{j}\right),$$

(24) $$i_{S-aln}\left(t\right)=s\left(t\right)\frac{R\sqrt{P_{S}S_{aln}\delta v}}{2}\sum_{i=-M}^{M}\cos\left[2\pi i\delta vt+\Delta \varphi_{iS}\right],$$

(25) $$i_{Ialn-LO}\left(t\right)=R\frac{\sqrt{P_{LO}S_{aln}\delta v}}{2}\sum_{i=-M}^{M}\cos\left[\left(\Delta \omega +2\pi i\delta v\right)t+\Delta \varphi_{iLO}\right],$$

(26) $$i_{Qaln-LO}\left(t\right)=R\frac{\sqrt{P_{LO}S_{aln}\delta v}}{2}\sum_{i=-M}^{M}\sin\left[\left(\Delta \omega +2\pi i\delta v\right)t+\Delta \varphi_{iLO}\right],$$

where $n_{0c}\left(t\right)$, $n_{90c}\left(t\right)$, $n_{180c}\left(t\right)$, and $n_{270c}\left(t\right)$ are additive noise of the four current signals, respectively; *i* and j represent the serial number of each frequency interval, respectively; $M=B_{o}/2\delta v$; $\Delta \omega =\omega_{S}-\omega_{LO}$; $\Delta \varphi =\varphi_{S}\left(t\right)-\varphi_{LO}\left(t\right)$; $\Delta \varphi_{iS}=\varphi_{i}-\varphi_{S}\left(t\right)$; $\Delta \varphi_{iLO}=\varphi_{i}-\varphi_{LO}\left(t\right)$.

From Equations (19)–(22), it is observed that the AL–AL beat noise and the signal light–AL beat noise are common-mode noises. They can be effectively eliminated by subtraction. However, the AL–LO beat noise is a differential-mode noise, that cannot be reduced by subtraction.

The current signals output by the I-channel BD and the Q-channel BD can be expressed as follows

(27) $$\begin{array}{l} i_{I}\left(t\right)=i_{0}\left(t\right)-i_{180}\left(t\right)=s\left(t\right)R\sqrt{P_{S}P_{LO}}\cos\left(\Delta \omega t+\Delta \varphi \right)\\ +R\sqrt{P_{LO}S_{aln}\delta v}\sum_{i=-M}^{M}\cos\left[\left(\Delta \omega +2\pi i\delta v\right)t+\Delta \varphi_{iLO}\right]+n_{Ic}\left(t\right) \end{array},$$

(28) $$\begin{array}{l} i_{Q}\left(t\right)=i_{90}\left(t\right)-i_{270}\left(t\right)=s\left(t\right)R\sqrt{P_{S}P_{LO}}\sin\left(\Delta \omega t+\Delta \varphi \right)\\ +R\sqrt{P_{LO}S_{aln}\delta v}\sum_{i=-M}^{M}\sin\left[\left(\Delta \omega +2\pi i\delta v\right)t+\Delta \varphi_{iLO}\right]+n_{Qc}\left(t\right) \end{array},$$

where $n_{Ic}\left(t\right)=n_{0c}\left(t\right)-n_{180c}\left(t\right)$; $n_{Qc}\left(t\right)=n_{90c}\left(t\right)-n_{270c}\left(t\right)$.

From these two equations, when the signal light contains AL and the OOK coherent receiving system reaches the shot noise limit, the system noise is mainly AL–LO beat noise and shot noise. The corresponding double sideband PSD of cosine function $A\cos\left(2\pi f_{0}t+\theta \right)$ is expressed as

(29) $$S_{\cos}\left(f\right)=\frac{A^{2}}{4}\delta \left(f-f_{0}\right)+\frac{A^{2}}{4}\delta \left(f+f_{0}\right),$$

As the optical bandwidth of the AL is much larger than the detector electrical bandwidth, and the frequency difference between the light signal and the LO is small, the frequency component in AL that can generate beat current with signal light is symmetrical about the central frequency of the signal light. Similarly, the frequency component in AL that can generate beat current with LO is also symmetrical about the central frequency of the LO. Thus, the current expression of the AL–LO beat term of channel I can also be approximated as

(30) $$i_{aln-LO}\left(t\right)=R\sqrt{P_{LO}S_{aln}\delta v}\sum_{i=-M}^{M}\cos\left[2\pi i\delta vt+\Delta \varphi_{iLO}\right],$$

In Equation (30), each frequency has both positive and negative terms. Their phases are random and cannot be combined directly. They can only be the sum of the PSD. Thus, the SSPSD of the AL–LO beat noise of Channel I is expressed as

(31) $$S_{aln-LO}\left(f\right)=\frac{1}{2}R^{2}P_{LO}S_{aln}\times 2=R^{2}P_{LO}S_{aln},$$

The current variance can be calculated according to the SSPSD of the AL–LO beat noise and the noise bandwidth of the detector using Equation (32).

(32) $$\sigma_{Ialn-LO}^{2}=R^{2}P_{LO}S_{aln}B_{e},$$

where $B_{e}$ denotes the noise bandwidth of the detector. The current variance corresponding to the channel I shot noise is expressed as

(33) $$\sigma_{Is}^{2}=eR\left(s\left(t\right)P_{s}+S_{aln}B_{o}+P_{LO}\right)B_{e},$$

where e is the electronic charge and $B_{o}$ is the receiver optical bandwidth. In Equation (33), $P_{LO}$ is far greater than the other two terms. The ratio of AL–LO beat noise current variance and shot noise current variance can be expressed as

(34) $$ratio=R^{2}P_{LO}S_{aln}B_{e}/\left(eR\left(s\left(t\right)P_{S}+S_{aln}B_{o}+P_{LO}\right)B_{e}\right)\approx RS_{aln}/e,$$

There is only a phase difference of π/2 between channels Q and I. Thus, the AL–LO beat noise current variance and shot noise in channel Q can also be expressed as Equation (34). The SNR expressions for channels I and Q are the same. Thus, the coherent OOK sensitivity loss expression introduced by AL noise can be expressed as follows in conditions dominated by LO shot noise.

(35) $$SNRloss_{co}=10\times \log10\left(\frac{RS_{aln}+e}{e}\right),$$

Equation (35) represents the loss of SNR when the AL enters the coherent OOK receiving system. Due to the subtraction function of the BDs, the noise introduced is mostly AL–LO beat noise.

The influence of AL on a non-coherent OOK receiver was considered. In this study, we chose the EDFA, PIN, and optical filter to receive OOK signals non-coherently. The OOK signal light containing AL after passing through the EDFA can be expressed as

(36) $$\begin{array}{l} E_{EDFA}\left(t\right)=\sqrt{G}\left(s\left(t\right)\sqrt{2P_{S}}\cos\left(\omega_{S}t+\varphi_{S}\left(t\right)\right)+\sum_{i=-M}^{M}\sqrt{2S_{aln}\delta v}\cos\left[\left(\omega_{S}+2\pi i\delta v\right)t+\varphi_{i}\right]\right)\\ +\sum_{l=-M}^{M}\sqrt{2S_{ase}\delta v}\cos\left[\left(\omega_{S}+2\pi l\delta v\right)t+\varphi_{l}\right] \end{array},$$

where G is the gain of the EDFA; $S_{ase}$ is the PSD of the ASE of the EDFA, considered to be flat within the optical bandwidth of the system; *l* represents the serial number of each frequency interval of the ASE; and $\varphi_{l}$ represents the random phase at each frequency interval of the ASE. As the optical bandwidths of the ASE and AL are often larger than the optical bandwidth of the system, it is considered that the ASE and AL noise have the same FWHM after filtering. The light output by the EDFA enters the PIN and is converted into the current signal, as is shown in the following

(37) $$\begin{array}{l} i_{preook}\left(t\right)=RGs^{2}\left(t\right)P_{S}+RGS_{aln}\delta v\sum_{i=-M}^{M}\sum_{j=-M}^{M}\cos\left(2\pi \left(i-j\right)\delta vt+\varphi_{i}-\varphi_{j}\right)+\\ RS_{ase}\delta v\sum_{l=-M}^{M}\sum_{n=-M}^{M}\cos\left(2\pi \left(l-n\right)\delta vt+\varphi_{l}-\varphi_{n}\right)+\\ 2RGs\left(t\right)\sqrt{P_{S}S_{aln}\delta v}\sum_{i=-M}^{M}\cos\left[\left(2\pi i\delta v\right)t+\Delta \varphi_{iS}\right]+\\ 2Rs\left(t\right)\sqrt{GP_{S}S_{ase}\delta v}\cos\sum_{l=-M}^{M}\cos\left[\left(2\pi l\delta v\right)t+\Delta \varphi_{Sase}\right]+\\ 2R\sqrt{GS_{aln}S_{ase}}\delta v\sum_{i=-M}^{M}\sum_{l=-M}^{M}\cos\left(2\pi \left(i-l\right)\delta vt+\varphi_{i}-\varphi_{l}\right)+i_{S}+i_{T} \end{array},$$

where $i_{S}$ represents the shot noise current; $i_{T}$ represents the thermal noise current; n represents the serial number of each frequency interval; and $\Delta \varphi_{Sase}=\varphi_{S}\left(t\right)-\varphi_{l}$. The first term in Equation (37) represents the communication signal that directly transmits the information. The main noise was caused by the cross terms.

The fourth term in Equation (37) represents the signal light–AL beat noise and its current variance can be obtained by its SSPSD and the detector noise bandwidth as expressed as

(38) $$\sigma_{IS-aln}^{2}=2R^{2}G^{2}P_{S}S_{aln}B_{e},$$

The fifth term in Equation (37) represents the signal light–ASE beat noise and its current variance can also be obtained by its SSPSD and the detector noise bandwidth as expressed as

(39) $$\sigma_{IS-ase}^{2}=2R^{2}GP_{S}S_{ase}B_{e},$$

The second term in Equation (37) represents the AL–AL beat noise. When *i* = *j*, a total of 2M DC terms are added to obtain Equation (40).

(40) $$i_{aln-aln}^{DC}=RGS_{aln}\delta v\times 2M=RGS_{aln}B_{o}=RGP_{aln},$$

where $P_{aln}$ is the AL power.

For each negative frequency component, there is always a corresponding positive frequency component: all negative frequencies correspond to the positive frequency range $\left(0,B_{o}\right)$. The corresponding positive frequency component multiplied by two is equivalent to the PSD multiplied by four. According to probability statistics, the PSD of the AL–AL beat noise decreases linearly from zero frequency to $B_{o}$, and its shape is a triangle.

The SSPSD close to zero frequency is expressed as

(41) $$S_{aln-aln}\left(\delta v\right)=4\times {\left(RGS_{aln}\delta v\right)}^{2}\left(\frac{B_{o}}{\delta v}-1\right)\times \frac{1}{2}/\delta v=2R^{2}G^{2}S_{aln}^{2}B_{o}=2R^{2}G^{2}S_{aln}P_{aln},$$

The SSPSD of other frequencies can be expressed as

(42) $$S_{aln-aln}\left(f\right)=2R^{2}G^{2}S_{aln}^{2}\left(B_{0}-f\right),$$

**Figure 11:**
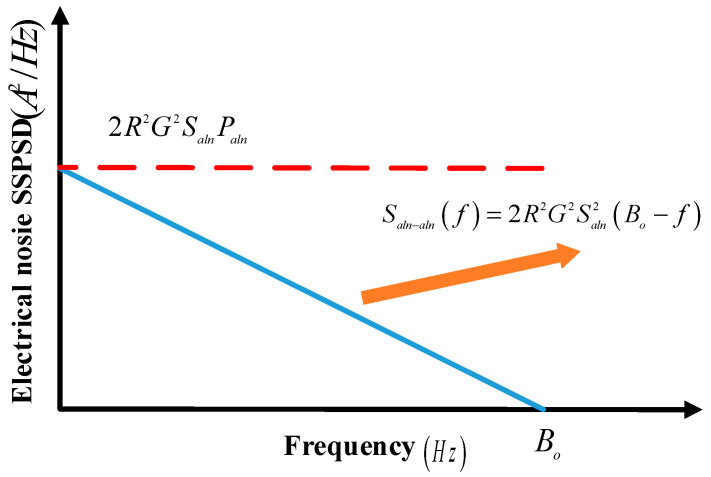
SSPSD of AL–AL beat noise.

The SSPSD of AL–AL beat noise is shown in Figure 11.

According to the noise bandwidth $B_{e}$ of the detector and the SSPSD of the AL–AL light beat noise, the AL–AL noise current variance is expressed as

(43) $$\sigma_{Ialn-aln}^{2}=\int_{0}^{B_{e}}S_{aln-aln}\left(f\right)df=R^{2}G^{2}S_{aln}^{2}B_{e}\left(2B_{o}-B_{e}\right),$$

The third term in Equation (37) represents the ASE–ASE beat noise. According to the analysis of the SSPSD of the AL–AL beat noise, the ASE–ASE beat noise current variance is expressed as

(44) $$\sigma_{Iase-ase}^{2}=R^{2}S_{ase}^{2}B_{e}\left(2B_{o}-B_{e}\right),$$

The sixth term in Equation (37) represents the AL–ASE beat noise. According to the analysis of the SSPSD of the AL–AL beat noise, the AL–ASE beat noise current variance is expressed

(45) $$\sigma_{Ialn-ase}^{2}=4R^{2}GS_{aln}S_{ase}B_{e}\left(2B_{o}-B_{e}\right),$$

The SSPSD and current variances of different noise for OOK signals containing AL received non-coherently with the preamplifier have been presented in a previous study. However, with the many noise types, sensitivity deterioration caused by AL should be further investigated through experiments.

## 5. Experimental Research on the Influence of AL on Coherent/Non-Coherent Reception of OOK Signal Light

### 5.1. AL Researh on the Coherent OOK Receiver

**Figure 12:**
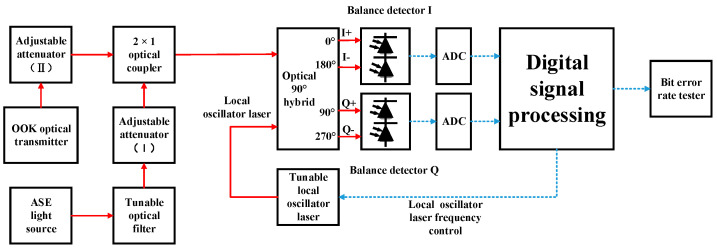
Experimental structure of the influence of AL on OOK coherent reception.

The experimental structure of the influence of AL on OOK coherent reception is shown in Figure 12.

In the coherent OOK reception experiment, channels I and Q have the same SNR; thus, the SNR of channel I is equivalent to the SNR of the entire system. This experiment analyzed the SNR of channel I to evaluate the impact of AL. In the experiment, an ASE light source was used to simulate AL. In the following description, we refer to the light output by the ASE light source as the simulated ambient light (SAL).

The LO power was 12.8 mW and the corresponding I-channel noise voltage variance is expressed as follows. The voltage variance is equal to the current variance multiplied by the square of the detector impedance.

(46) $$\sigma_{Vs}^{2}\approx eRP_{LO}B_{e}r^{2}=1.088\times 10^{-4}\;\left(V^{2}\right),$$

where $B_{e}$ is the noise bandwidth of the BDs, $B_{e}=2.5\;\mathrm{GHz}$; r is the detector impedance, r=5 kΩ.

In this experiment, the wavelength range of the ASE light source used was 1526.65 nm to 1570.85 nm. The tunable optical filter was adjusted, such that its central wavelength was equal to the signal optical wavelength (1555.74 nm): the bandwidth was 2 nm, from 1554.74 nm to 1556.74 nm. The frequency interval corresponding to the wavelength interval was $2.48\times 10^{11}\;\mathrm{Hz}$. By adjusting the attenuator, the SAL powers entering the 2 × 1 coupler were 1.15 μW, 0.86 μW, 0.58 μW, 0.29 μW, 0.14 μW, 57.56 nW, 46.05 nW, 28.78 nW, and 5.76 nW. The PSD of the SAL was considered to be flat in the filter passband. According to the power and frequency intervals, we can obtain the corresponding PSD of the SAL with different powers, as shown in Table 2.

Equation (47) was used for calculations in the third column of Table 2.

(47) $$\sigma_{Valn-lo}^{2}=R^{2}P_{LO}S_{aln}r^{2}B_{e},$$

In this experiment, before adding the SAL, the voltage variance of the shot noise was the main noise component. Adding SAL with different PSDs can yield new voltage noise variances. The new voltage variance consists of shot noise and AL–LO beat noise; both are white noise. The ratio of the new voltage noise variance to the shot noise variance represents the change in noise, and the deterioration of system sensitivity. The calculated and experimentally measured ratios are presented in Table 3. Equation (35) was used for calculations in the second column of Table 3. Actual measured data were calculated and used for the third column of Table 3.

The measured ratio was smaller than the theoretical ratio because the measured value of the voltage variance of the shot noise was larger than the theoretical value. This may have been due to oscilloscope quantization error.

**Figure 13:**
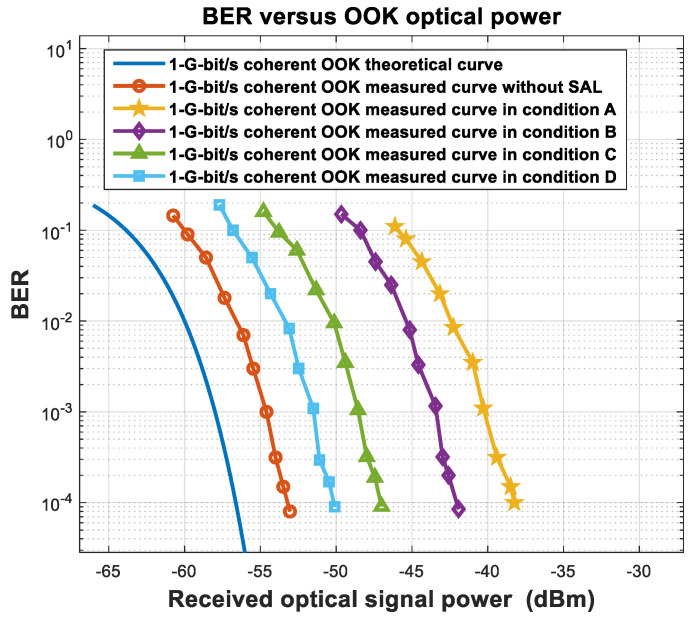
BER vs. coherent OOK optical power in different conditions.

We tested the sensitivity in four conditions: A, B, C, and D (Table 3), and coherently received OOK signals at a communication rate of 1 Gbit/s. The relationship curves for coherent reception between the BER and the optical signal power in different conditions are shown in Figure 13.

Figure 13 shows the theoretical curve, the curve without SAL, and the curves in conditions D, C, B, and A. When the BER is 10^−3^, the deterioration in sensitivity of D, C, B, and A was 14.3 dB, 11.3 dB, 6.1 dB, and 3.09 dB, respectively, consistent with the theoretical calculation in Table 3. The noise bandwidth of the BD in this experiment was 2.5 GHz. The SAL noise outside the band did not increase SAL–LO beat noise voltage variance, but increased part of the shot noise. However, the power of the LO was dominant; the shot noise generated by the SAL can be ignored. When the filter bandwidth was 0.2 nm–5 nm, the noise voltage variance was essentially the same as that at 2 nm, with the PSD of the SAL and the central wavelength of the optical filter unchanged.

### 5.2. AL Researh on the Non-Coherent OOK Receiver

**Figure 14:**
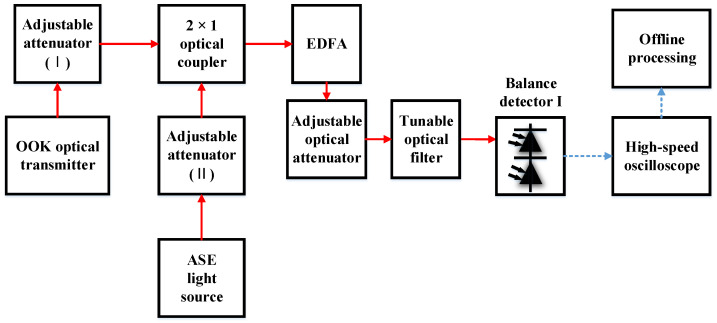
Experimental structure diagram of the influence of AL on OOK non-coherent reception.

The experimental structure of the influence of AL on OOK non-coherent reception is shown in Figure 14.

The central wavelength of the tunable filter was set as the optical signal wavelength, and the FWHM of the filter passband was set to 0.8 nm. The wavelength of the SAL output from the ASE light source ranged from 1526.65 nm–1570.85 nm, corresponding to a frequency of $5.53\times 10^{12}\;\mathrm{Hz}$. The SAL power was set to 1.03 μW, 3.29 μW, 6.58 μW, 12.84 μW, 25.68 μW, and 51.35 μW. With the EDFA, an additional 50% power loss was introduced, caused by the polarizer. The SAL power and the PSD of the SAL entering the non-coherent OOK system are shown in Table 4. In addition, we use letters that do not appear to represent the different experimental conditions, as shown in the first column of Table 4.

**Figure 15:**
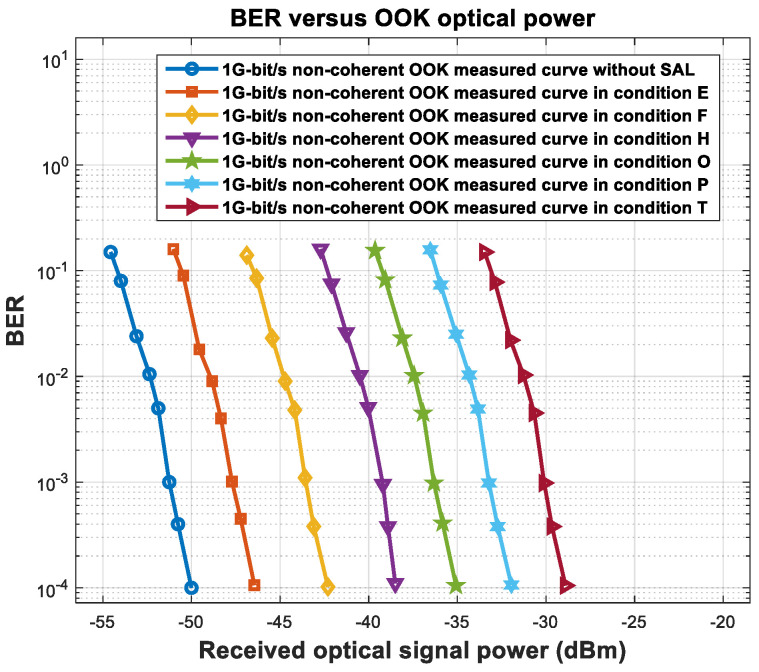
BER vs. non-coherent OOK optical power in different conditions.

We tested the sensitivity in six conditions (E, F, H, O, P, T) and non-coherently received OOK signals at a communication rate of 1 Gbit/s. The relationship curves for non-coherent reception between the BER and the optical signal power in different conditions are shown in Figure 15.

### 5.3. Analysis and Discussion of the Experimental Results

**Figure 16:**
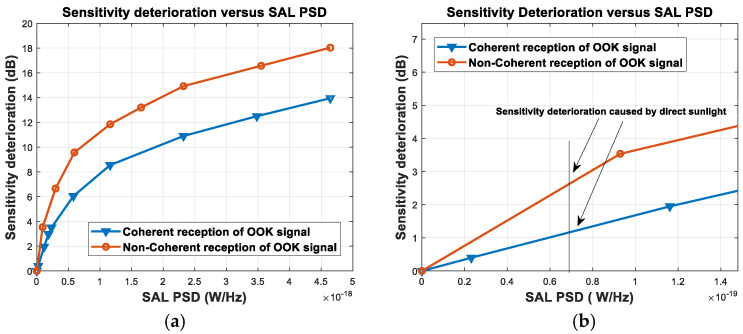
Curves of sensitivity deterioration vs. SAL PSD ((**a**) global and (**b**) local).

From Figure 13 and Figure 15, the relationship curves between the SAL PSD and the amount of sensitivity deterioration for coherent and non-coherent reception are shown in Figure 16.

The PSD of the sunlight coupled to the receiver SMF was $6.84\times 10^{-20}\;W/\mathrm{Hz}$ at a central wavelength of 1555.74 nm. From the curves of coherent reception and non-coherent reception in Figure 16, it can be concluded that the sunlight power coupled into the receiver SMF caused 1.15 dB sensitivity deterioration to the coherent OOK receiver and 2.60 dB sensitivity deterioration to the non-coherent OOK receiver when the sun shines directly. These results are similar to those reported by Fidler et al. [46]. Thus, the coherent OOK receiving system has better anti-AL noise performance than the non-coherent OOK receiving system, and the coherent and non-coherent OOK receivers requiring SMF coupling can both work in direct sunlight.

## 6. Conclusions

In this study, an FSO OOK coherent/non-coherent communication receiver was built. To improve receiver sensitivity, non-coherent receiving mode with EDFA and coherent receiving mode without EDFA were selected. With a communication rate of 1 Gbit/s and a BER of 10^−3^, the optical power required by the coherent and non-coherent systems were −54.60 dBm and −51.25 dBm, respectively, both with high sensitivity. The coherent mode was 3.3 dB from the shot noise limit. The influence of AL on OOK coherent and non-coherent receiving systems was theoretically analyzed. For a coherent OOK system, the AL–LO beat noise was mainly introduced by AL. When the AL is sufficiently large, the AL–LO beat noise exceeds the shot noise and dominates. For a non-coherent OOK receiving system with a preamplifier, AL causes new noise. The SSPSD of this noise and the corresponding current variance were reported. An ASE light source was used to simulate AL. The influence of SAL on the OOK coherent and non-coherent receiving systems was experimentally investigated. The relationship curves between the BER and received optical power were presented for different SAL PSDs using the two systems. The experimental results of the influence of AL on the coherent OOK receiving system were consistent with the theoretical results. The optical bandwidth was set to 2 nm for coherent reception and 0.8 nm for non-coherent reception. The experiment demonstrates that the OOK coherent receiver has better anti-AL noise performance than the OOK non-coherent receiver. The experiment was based on the verification completed by the optical fiber system. The main index used to evaluate the intensity of AL was the PSD of the AL (W/Hz). To better relate the experimental results with an actual FSO system, the link margin of the FSO system was calculated. Using the sun as the main noise source of the FSO system, the average coupling efficiency of the sunlight to the receiver SMF was analyzed. When the receiver lens diameter was greater than 0.488 mm, the power coupled into the receiver SMF did not increase with an increase in the receiver lens diameter, essentially remaining the same, indicating that AL noise can be suppressed to a certain extent by increasing the receiver lens diameter because the receiver gain increased with an increase in the receiver lens diameter. When the sun shines directly and the receiver lens diameter is greater than 0.488 mm, the coherent OOK receiving system coupled with the SMF and the non-coherent receiving system coupled with the SMF produce sensitivity losses of 1.15 dB and 2.60 dB, respectively, demonstrating that the FSO system can work in direct sunlight. When the BPSK signal or QPSK signal is coherently received, the change in dominant noise caused by AL noise on the corresponding coherent receiver is consistent with that of the OOK coherent receiver. This study is of great significance to the further study of the influence of AL noise on FSO receivers in the future.

## Figures and Tables

**Table 1:** Key experimental parameters.

Experimental Parameters	Symbol	Value
Laser wavelength (signal and LO light)	λ	1555.74 nm
Laser linewidth (signal and LO light)	Δv	5000 Hz
Communication rate	$R_{b}$	1 Gbit/s
Received optical signal power	$P_{S}$	−65 to −30 dBm
Detector responsivity	R	0.85 A/W
Detector transimpedance	r	5 kΩ
Optical 90° hybrid splitting ratio	$k_{S}\&k_{L0}$	0.5

**Table 2:** SAL power, PSD of SAL, and SAL–LO beat noise voltage variance (coherent).

SAL Power	PSD of SAL	Voltage Variance of SAL–LO Beat Noise
1.15 μW	$4.64\times 10^{-18}\;W/\mathrm{Hz}$	$2.70\times 10^{-3}{\;V}^{2}$
0.86 μW	$3.48\times 10^{-18}\;W/\mathrm{Hz}$	$2.00\times 10^{-3}{\;V}^{2}$
0.58 μW	$2.32\times 10^{-18}\;W/\mathrm{Hz}$	$1.30\times 10^{-3}{\;V}^{2}$
0.29 μW	$1.16\times 10^{-18}\;W/\mathrm{Hz}$	$6.71\times 10^{-4}{\;V}^{2}$
0.14 μW	$5.80\times 10^{-19}\;W/\mathrm{Hz}$	$3.36\times 10^{-4}{\;V}^{2}$
57.56 nW	$2.32\times 10^{-19}\;W/\mathrm{Hz}$	$1.34\times 10^{-4}{\;V}^{2}$
46.05 nW	$1.86\times 10^{-19}\;W/\mathrm{Hz}$	$1.07\times 10^{-4}{\;V}^{2}$
28.78 nW	$1.16\times 10^{-19}\;W/\mathrm{Hz}$	$6.71\times 10^{-5}{\;V}^{2}$
5.76 nW	$2.32\times 10^{-20}\;W/\mathrm{Hz}$	$1.34\times 10^{-5}{\;V}^{2}$

**Table 3:** PSD of SAL, ratio of new noise variance to shot noise variance (theory and experiment).

PSD of SAL	Ratio of New Noise Variance to Shot Noise Variance (Theory)	Ratio of New Noise Variance to Shot Noise Variance (Experiment)
$4.64\times 10^{-18}\;W/\mathrm{Hz}$ (A)	14.09 dB	13.90 dB
$3.48\times 10^{-18}\;W/\mathrm{Hz}$	12.90 dB	12.35 dB
$2.32\times 10^{-18}\;W/\mathrm{Hz}$ (B)	11.25 dB	10.45 dB
$1.16\times 10^{-18}\;W/\mathrm{Hz}$	8.55 dB	8.16 dB
$5.80\times 10^{-19}\;W/\mathrm{Hz}$ (C)	6.11 dB	5.97 dB
$2.32\times 10^{-19}\;W/\mathrm{Hz}$	3.49 dB	3.17 dB
$1.86\times 10^{-19}\;W/\mathrm{Hz}$ (D)	2.98 dB	2.76 dB
$1.16\times 10^{-19}\;W/\mathrm{Hz}$	2.09 dB	1.90 dB
$2.32\times 10^{-20}\;W/\mathrm{Hz}$	0.51 dB	0.31 dB

**Table 4:** SAL power, actual PSD of SAL (non-coherent).

SAL Power	Actual PSD of SAL
1.03 μW (E)	$9.29\times 10^{-20}\;W/\mathrm{Hz}$
3.29 μW (F)	$2.98\times 10^{-19}\;W/\mathrm{Hz}$
6.58 μW (H)	$5.95\times 10^{-19}\;W/\mathrm{Hz}$
12.84 μW (O)	$1.16\times 10^{-18}\;W/\mathrm{Hz}$
25.68 μW (P)	$2.32\times 10^{-18}\;W/\mathrm{Hz}$
51.35 μW (T)	$4.64\times 10^{-18}\;W/\mathrm{Hz}$

## Data Availability

No applicable.

## References

[1] Kaushal H., Kaddoum G. (2017). Optical Communication in Space: Challenges and Mitigation Techniques. IEEE Commun. Surv. Tutor..

[2] Oppenhauser G., Wittig M. European SILEX Project: Concept, Performance, Status, and Planning. Proceedings of the Free-Space Laser Communication Technologies II.

[3] Tolker-Nielsen T., Oppenhauser G. In-orbit test result of an operational optical intersatellite link between ARTEMIS and SPOT4, SILEX. Proceedings of the Free-Space Laser Communication Technologies XIV.

[4] Toyoshima M., Yamakawa S., Yamawaki T., Arai K., Reyes M., Alonso A., Sodnik Z., Demelenne B. Ground-to-satellite optical link tests between Japanese laser communications terminal and European geostationary satellite ARTEMIS. Proceedings of the Free-Space Laser Communication Technologies XVI.

[5] Jono T., Takayama Y., Kura N., Ohinata K., Koyama Y., Shiratama K., Sodnik Z., Demelenne B., Bird A., Arai K. OICETS on-orbit laser communication experiments. Proceedings of the Free-Space Laser Communication Technologies XVIII.

[6] Boroson D., Robinson B., Murphy D., Burianek D., Khatri F., Kovalik J., Sodnik Z., Cornwell D. Overview and results of the Lunar Laser Communication Demonstration. Proceedings of the Free-Space Laser Communication and Atmospheric Propagation XXVI.

[7] Boroson D.M., Scozzafava J.J., Murphy D.V., Robinson B.S., Shaw H. The Lunar Laser Communications Demonstration (LLCD). Proceedings of the Smc-It 2009: Third Ieee International Conference on Space Mission Challenges for Information Technology.

[8] Robinson B.S., Boroson D.M., Burianek D.A., Murphy D.V. The lunar laser communications demonstration. Proceedings of the 2011 International Conference on Space Optical Systems and Applications (ICSOS).

[9] Yamakawa S., Chishiki Y., Sasaki Y., Miyamoto Y., Kohata H. JAXA’s optical data relay satellite programme. Proceedings of the 2015 IEEE International Conference on Space Optical Systems and Applications (ICSOS).

[10] Chen W., Sun J., Hou X., Zhu R., Hou P., Yang Y., Gao M., Lei L., Xie K., Huang M. 5.12 Gbps optical communication link between LEO satellite and ground station. Proceedings of the 2017 IEEE International Conference on Space Optical Systems and Applications (ICSOS).

[11] Fong T.K., Sabido D.J., Kalman R.F., Tabara M., Kazovsky L.G. (1994). Linewidth-insensitive coherent AM optical links: Design, performance, and potential applications. J. Light. Technol..

[12] Becker D., Wree C., Mohr D., Joshi A. Unpreamplified heterodyne detection of 10Gb/s NRZ-OOK with high receiver sensitivity. Proceedings of the Optical Transmission, Switching, and Subsystems IV.

[13] Wree C., Becker D., Mohr D., Joshi A. (2007). Measured noise performance for heterodyne detection of 10-Gb/s OOK and DPSK. IEEE Photonics Technol. Lett..

[14] Li M., Tan L., Ma J., Yu S., Wu J., Wang Q. Performance analysis of OOK receiver with a GSM laser in space to ground optical communication link. Proceedings of the Laser Communication and Propagation through the Atmosphere and Oceans IV.

[15] Li M., Li T., Zhang X., Song Y., Liu Y. Bit error rate analysis of ook modulation scheme under non-coherent demodulation for space uplink optical communication systems. Proceedings of the 2015 IEEE 16th International Conference on Communication Technology (ICCT).

[16] Parween S., Tripathy A. Free space optic communication using optical AM, OOK-NRZ and OOK-RZ modulation techniques. Proceedings of the 2019 3rd International Conference on Electronics, Materials Engineering & Nano-Technology (IEMENTech).

[17] You Q., Chen D., Xiao X., Yu S. (2021). 10 Gb/s free space optical interconnect with broadcasting capability enabled by a silicon integrated optical phased array. Chin. Opt. Lett..

[18] Yeh C.-H., Chow C.-W., Wei L.-Y. (2019). 1250 Mbit/s OOK wireless white-light VLC transmission based on phosphor laser diode. IEEE Photonics J..

[19] Lu Z., Tian P., Chen H., Baranowski I., Fu H., Huang X., Montes J., Fan Y., Wang H., Liu X. (2017). Active tracking system for visible light communication using a GaN-based micro-LED and NRZ-OOK. Opt. Express.

[20] Li Y., Li M., Poo Y., Ding J., Tang M., Lu Y. (2014). Performance analysis of OOK, BPSK, QPSK modulation schemes in uplink of ground-to-satellite laser communication system under atmospheric fluctuation. Opt. Commun..

[21] Xu Z., Xu G., Zheng Z. (2021). BER and channel capacity performance of an FSO communication system over atmospheric turbulence with different types of noise. Sensors.

[22] Li H., Sang X. (2017). SNR and transmission error rate for remote laser communication system in real atmosphere channel. Sens. Actuators A Phys..

[23] Trinh P.V., Dang N.T., Pham A.T. (2015). All-optical relaying FSO systems using EDFA combined with optical hard-limiter over atmospheric turbulence channels. J. Light. Technol.

[24] Bosu R., Prince S. (2019). Mitigation of turbulence induced scintillation using concave mirror in reflection-assisted OOK free space optical links. Opt. Commun..

[25] Toyoshima M., Fukazawa T., Toyoda M., Shikatani M., Takahashi T., Araki K., Arimoto Y., Aruga T. Measurements of background noise from the earth surface using the ETS-VI/LCE. Proceedings of the Free-Space Laser Communication Technologies VIII.

[26] Fu Q., Liu P., Tong S., Zhang P. Study on the effects of the space environment on laser transmission characteristics. Proceedings of the International Symposium on Optoelectronic Technology and Application 2014: Optical Remote Sensing Technology and Applications.

[27] Lu W., Sun J., Hou P., Xu Q., Xi Y., Zhou Y., Zhu F., Liu L. Multiple wavelength spectral system simulating background light noise environment in satellite laser communications. Proceedings of the Laser Communication and Propagation through the Atmosphere and Oceans VI.

[28] Farrell T.C. Sources of background light on space based laser communications links. Proceedings of the Sensors and Systems for Space Applications XI.

[29] Yan X., Cao C., Zhang W., Zeng X., Feng Z., Wu Z., Wang T. (2021). Wavefront Coherent Compensation Technology Under Direct Sunlight in Free Space Optical Communication System. IEEE Photonics J..

[30] Giggenbach D. (2022). Free-Space Optical Data Receivers with Avalanche Detectors for Satellite Downlinks Regarding Background Light. Sensors.

[31] Yariv A. (1990). Signal-to-noise considerations in fiber links with periodic or distributed optical amplification. Opt. Lett..

[32] Olsson N.A. (1989). Lightwave systems with optical amplifiers. J. Light. Technol..

[33] Chen G., Yin F. High-speed long-wavelength APDs for optical communication. Proceedings of the Advanced Materials and Devices for Sensing and Imaging.

[34] Barry J.R., Lee E.A. (1990). Performance of coherent optical receivers. Proc. IEEE.

[35] Derr F. (1992). Coherent optical QPSK intradyne system: Concept and digital receiver realization. J. Light. Technol..

[36] Garreis R.B. 90 degree optical hybrid for coherent receivers. Proceedings of the Optical Space Communication II.

[37] Lu S., Zhou Y., Zhu F., Sun J., Yang Y., Zhu R., Hu S., Zhang X., Zhu X., Hou X. (2020). Digital-analog hybrid optical phase-lock loop for optical quadrature phase-shift keying. Chin. Opt. Lett..

[38] Wu Q., Zhu Y., Cheng Z., Yin L., Hu W. (2022). Spectrally sliced heterodyne coherent receiver with halved electrical bandwidth. Chin. Opt. Lett..

[39] Okoshi T. (1982). Heterodyne and coherent optical fiber communications: Recent progress. IEEE Trans. Microw. Theory Tech..

[40] Ren W., Sun J., Hou P., Han R., He H., Cong H., Li C., Zhang L., Jiang Y. (2022). Direct phase control method for binary phase-shift keying space coherent laser communication. Chin. Opt. Lett..

[41] Sowailem M.Y., Hoang T.M., Morsy-Osman M., Chagnon M., Patel D., Paquet S., Paquet C., Woods I., Liboiron-Ladouceur O., Plant D.V. (2016). 400-G single carrier 500-km transmission with an InP dual polarization IQ modulator. IEEE Photonics Technol. Lett..

[42] Ruilier C., Cassaing F. (2001). Coupling of large telescopes and single-mode waveguides: Application to stellar interferometry. JOSA A.

[43] Ma J., Zhao F., Tan L., Yu S., Han Q. (2009). Plane wave coupling into single-mode fiber in the presence of random angular jitter. Appl. Opt..

[44] Zhao F., Ma J., Yu S., Tan L., Han Q. (2010). Impact of random angular jitter on fiber-coupled differential phase-shift keying receivers with Mach–Zehnder interferometer demodulation. Appl. Opt..

[45] Lavenda B., Dunning-Davies J. (1990). Stefan-Boltzmann law for black bodies and black holes. Int. J. Theor. Phys..

[46] Fidler F., Wallner O. (2008). Application of single-mode fiber-coupled receivers in optical satellite to high-altitude platform communications. EURASIP J. Wirel. Commun. Netw..

